# The value of a risk model combining specific risk factors for predicting postoperative severe morbidity in biliary tract cancer

**DOI:** 10.3389/fonc.2023.1309724

**Published:** 2024-02-05

**Authors:** BaoLong Ye, JunFeng Xie, KeXing Xi, ZhiShun Huang, YanNian Liao, ZiWen Chen, Wu Ji

**Affiliations:** ^1^ Department of Gastrointestinal and Hernia Surgery, Ganzhou Hospital-Nanfang Hospital, Southern Medical University, Ganzhou, China; ^2^ Department of General Surgery, Nanfang Hospital, Southern Medical University, Guangzhou, China; ^3^ Department of Gastrointestinal and Hernia Surgery, The Affiliated Ganzhou Hospital of Nanchang University, Ganzhou, China; ^4^ Research Institute of General Surgery, Jinling Hospital, Medical School of Nanjing University, Nanjing, China

**Keywords:** morbidity, risk prediction models, risk score, specific risk factors, biliary tract cancer

## Abstract

**Purpose:**

Several surgical risk models are widely utilized in general surgery to predict postoperative morbidity. However, no studies have been undertaken to examine the predictive efficacy of these models in biliary tract cancer patients, and other perioperative variables can also influence morbidity. As a result, the study’s goal was to examine these models alone, as well as risk models combined with disease-specific factors, in predicting severe complications.

**Methods:**

A retrospective study of 129 patients was carried out. Data on demographics, surgery, and outcomes were gathered. These model equations were used to determine the morbidity risks. Severe morbidity was defined as the complication comprehensive index ≥ 40.

**Results:**

Severe morbidity was observed in 25% (32/129) patients. Multivariate analysis demonstrated that four parameters [comprehensive risk score ≥1, T stage, albumin decrease value, and international normalized ratio (INR)] had a significant influence on the probability of major complications. The area under the curve (AUC) of combining the four parameters was assessed as having strong predictive value and was superior to the Estimation of Physiologic Ability and Surgical Stress System (E-PASS) alone (the AUC value was 0.858 *vs.* 0.724, p = 0.0375). The AUC for the modified E-PASS (mE-PASS) and Physiological and Operative Severity Score for the Enumeration of Mortality and Morbidity (POSSUM) in patients over the age of 70 was classified as no predictive value (p = 0.217 and p = 0.063, respectively).

**Conclusion:**

The mE-PASS and POSSUM models are ineffective in predicting postoperative morbidity in patients above the age of 70. In biliary tract cancer (BTC) patients undergoing radical operation, a combination of E-PASS and perioperative parameters generates a reasonable prediction value for severe complications.

## Background

Currently, radical surgical resection is the only way to cure biliary tract cancer (BTC), which includes intrahepatic, perihilar, distal cholangiocarcinoma, and gallbladder cancer, as well as ampulla of Vater cancer. However, because of the high risk of severe complications, performing a radical operation is usually challenging. Operation risks are increasing due to the need for biliary tract reconstruction and, in some cases, resecting and reconstructing the hepatic artery and portal vein, as well as pancreatoduodenectomy (PD) (1). As a result, creating and utilizing an accurate and effective preoperative evaluation approach are helpful in boosting operation safety, minimizing risks, and improving BTC patients’ quality of life.

In recent years, several morbidity classification systems have established a method for measuring morbidity, including the widely used Clavien–Dindo classification system (CDC). However, these systems take into account severe morbidity but exclude other serious relevant events, whereas BTC patients undergoing radical surgery may experience several complications (2, 3). The common complications after radical surgery for patients with BTC are postoperative biliary fistula, postoperative hemorrhage, intra-abdominal abscess, and acute cholangitis, which can occur during a variety of surgical operations related to hepatobiliary and pancreatic surgeries (4, 5). Postoperative complications continue to be the main factor of increased morbidity and mortality, frequently resulting in a longer hospital stay, delayed removal of abdominal drains, and the need for extra diagnostic tests or procedures (6). For example, bile leakage is officially defined by the International Study Group of Liver Surgery (ISGLS) as a discharge of fluid with an increased bilirubin concentration *via* intra-abdominal drains on or after postoperative Day 3 or as the need for radiologic intervention (i.e., interventional drainage) and relaparotomy for biliary collections and bile peritonitis, respectively (5). In 2013, a new classification system was presented to address this issue, named the Comprehensive Complication Index (CCI). The CCI is used to assess the cumulative effect of every postoperative adverse result in a single patient, ranging from 0 (no morbidity) to 100 (death). This grading system has previously been shown to be effective in biliary cancer patients (7). Estimation of Physiologic Ability and Surgical Stress System (E-PASS) and six variables reflecting patient physical conditions (PRS) and three variables reflecting surgery stress (SSS) together make up the comprehensive risk score (CRS). On this basis, the modified Estimation of Physiologic Ability and Surgical Stress System (mE-PASS) was created successfully by reducing SSS variables from 3 to 1. The Physiological and Operative Severity Score for the Enumeration of Mortality and Morbidity (POSSUM) consists of 12 physiology scores (PS) and six operational score (OS). Over the past few years, several studies have confirmed the effectiveness of these three models in predicting severe postoperative complications in general department surgery (8, 9). However, no studies comparing the predictive efficacy of these three models in BTC patients have been conducted, and morbidity can also be influenced by other perioperative variables. Therefore, the purpose of this study was to compare the E-PASS, mE-PASS, and POSSUM models alone in predicting major complications in BTC patients undergoing surgical resection, as well as estimating the predictive validity of the risk model associated with perioperative variables of severe adverse outcomes.

## Patients and methods

### Patients

Data were gathered from a database that contained full information on patients admitted to the Research Institute of General Surgery of Jinling Hospital for biliary tract cancer from 1 January 2015 to 31 August 2022. All patients undergoing radical resection were included in this study except those who had received adjuvant therapy before surgery or patients with a previous history of abdominal malignancy. Biliary malignancy was confirmed in all patients by pathological examination. Before surgery, every individual completed informed consent forms.

### Preoperative preparation

All patients were treated according to standard operating guidelines (10). Biliary drainage was performed prior to surgery in patients with cholangitis, prolonged biliary obstruction, and total bilirubin levels greater than 200 mol/L, and the target level of total bilirubin was less than 50 mol/L before surgery. If a patient’s future liver remnant (FLR) volume was greater than 50%, the target total bilirubin before surgery was allowed to exceed 50 mol/L (11). Depending on the patient’s condition, either percutaneous transhepatic biliary drainage (PTBD) or endoscopic nasobiliary drainage (ENBD) was used for biliary drainage. Patients with severe preoperative cholangitis were treated until the indications for infection disappeared.

### Surgical technique

Hepatectomy (segmentectomy, lobectomy, and extended lobectomy), cholecystectomy, extrahepatic bile duct resection with Roux-en-Y hepaticojejunostomy, and pancreaticoduodenectomy were the standard surgeries performed. Skeletonization of the hepatoduodenal ligament, including the lymph nodes surrounding the head of the pancreas, was regularly conducted in all kinds of biliary malignancies (12). In patients with vascular tumor invasion diagnosed after intraoperative evaluation, vascular resection and reconstruction were conducted. Combined pancreaticoduodenectomy was undertaken in some cases with pancreatic or duodenal invasion, and reconstruction was performed beginning with pancreaticojejunostomy anastomosis, followed by hepaticojejunostomy anastomosis and then extracorporeal duodenojejunostomy. Detailed descriptions of pancreaticoduodenectomy have been previously published (13, 14), and some individuals with no prior evidence of vascular invasion underwent total laparoscopy (11, 15, 16).

### Calculation of the risk score

The CRS is computed according to the E-PASS model, and CRSf is calculated according to the mE-PASS model as published previously. Briefly, CRS is derived by combining six preoperative variables and three surgical parameters; CRSf is generated based on the same PRS and surgical stress score fixed (SSSf). The calculation of the CRS, PRS, SSS, CRSf, and SSSf is as follows (9, 17):


$$\text{CRS }=\;-0.328\;+\;\left(0.936\;\times \text{ PRS}\right)\;+\;\left(0.976\;\times \text{ SSS}\right),$$

PRS = −0.0686 + 0.00345X1 + 0.323X2 + 0.205X3 + 0.153X4 + 0.148X5 + 0.0666X6,

where X1 represents age, X2 represents patients with or without severe heart disease (1 or 0 points), X3 represents patients with or without severe pulmonary disease (1 or 0 points), X4 represents patients with or without diabetes mellitus (1 or 0 points), X5 represents the index of performance condition (range, 0–4 points), and X6 represents the American Society of Anesthesiologists (ASA) score (range, 1–5 points). Severe heart disease was considered as New York Heart Association (NYHA) grade 3/4 or serious cardiac asthma needing mechanical assistance. Serious pulmonary illness was classified as a vital capacity of less than 60% or a first second of forced expiratory volume of less than 50%.


SSS = −0.342 + 0.0139X1 + 0.0392X2 + 0.352X3,

where X1 is the ratio of blood loss (ml) to weight (kg), X2 represents surgical duration (h), and X3 is the skin incision (minimally invasive surgery or laparotomy or laparotomy with thoracotomy indicating 0, 1, and 2, respectively).


$$\text{CRSf }=\;0.052\;+\;\left(0.58\;\times \text{ PRS}\right)\;+\;\left(0.83\;\times \text{ SSSf}\right).$$

The SSSf score is shown in **Table 1**.

**Table 1:** Eligible procedures and SSSf.

Main procedures	SSSf
Cholecystectomy for malignant tumor	0.309
Resection of common bile duct for malignant tumor	0.401
Hepatectomy	
1. Segmentectomy	0.453
2. Lobectomy	0.663
3. Extended lobectomy	1.025
Resection of pancreatic head tumor	
1. Pancreatoduodenectomy	0.496
2. Tumor resection with nodal dissection or resection of pancreatic head with duodenum preservation	0.612
3. Tumor resection with surrounding organs such as stomach, colon, kidney, and adrenal gland	1.028
4. Tumor resection with blood vessel reconstruction	1.028

SSSf, surgical stress score fixed.

Nineteen variables were used to calculate a PS and an OS. PS variables included age, cardiac signs, respiratory signs, pulse rate, systolic blood pressure, Glasgow coma score, serum urea, potassium, and sodium concentrations, hemoglobin concentration, white cell count, and electrocardiographic data. OS factors were the number of surgical operations, blood loss, peritoneal soiling, presence of malignancy, grade, and timing of surgery. Each variable was divided into four grades, with higher scores (1, 2, 4, and 8) reflecting higher levels. The POSSUM morbidity rate (R1) was calculated by inserting PS and OS into regression models (18), and the equation is as follows:


$$\text{lnR}1/\left(1\;-\text{ R}1\right)\;=\;-5.91\;+\;0.16\;\times \text{ PS }+\;0.19\;\times \text{ OS.}$$

### Perioperative factors

The preoperative factors assessed included age, gender, body mass index, tumor type, leucocyte count, ENBD or PTBD performed, preoperative total bilirubin level, Δalbumin (the reduced values between the first day after surgery and preoperation), Δhemoglobin (the reduced values between the first day after surgery and preoperation), international normalized ratio, T stage (Union for International Cancer Control (UICC) of 8th edition), preoperative cholangitis (Tokyo Guidelines 2018), minimally invasive surgery, and CRS ≥ 1. Earlier research has shown that CRS ≥ 1 is crucial in the occurrence of severe complications after elective gastrointestinal surgery (17).

### Definition of morbidity

Morbidity data were obtained from all patients’ medical records in the prospective database. Postoperative morbidity was recorded from the day of surgery until the patient was discharged, as well as when the patients were readmitted due to operation-related problems within 3 months after surgery. The CDC grading system (range, I–V) was used to evaluate and classify major postoperative complications (18). In addition, the CCI (range, 0–100) was computed online utilizing free access at www.assessurgery.com. Severe morbidity was classified in this study as CCI ≥ 40, which was characterized as having one or more life-threatening morbidities (CDC grade IV) (9). The ISGLS criteria were used to assess hepatic failure, intraperitoneal hemorrhage, and bile leakage (3, 19, 20). Cholangitis was classified into three severity levels according to Tokyo Guidelines 2018 (21).

### Statistical analysis

For each factor, univariate and multivariate analyses were utilized. When p< 0.30 was obtained by univariate analysis, the variables were subjected to multivariate analysis. Pearson’s χ^2^ test and the Mann–Whitney U test were used individually to compare binary and continuous data. The Hosmer–Lemeshow chi-square statistic was applied to assess the goodness of fit for comparing observed and projected outcomes at different risk deciles. Using MedCalc software, the predictive power of the perioperative variables identified in multivariate analyses was assessed by calculating the area under the curve (AUC) value of receiver operating characteristic (ROC) plots. AUC values ranging from 0.7 to 0.9 indicate good predictive value, while values less than 0.7 indicate poor predictive value. p< 0.05 was considered statistically significant. Data analysis was performed using SPSS software (version 25.0) and MedCalc software (version 11.4.2.0).

## Results

### Patients

**Figure 1:**
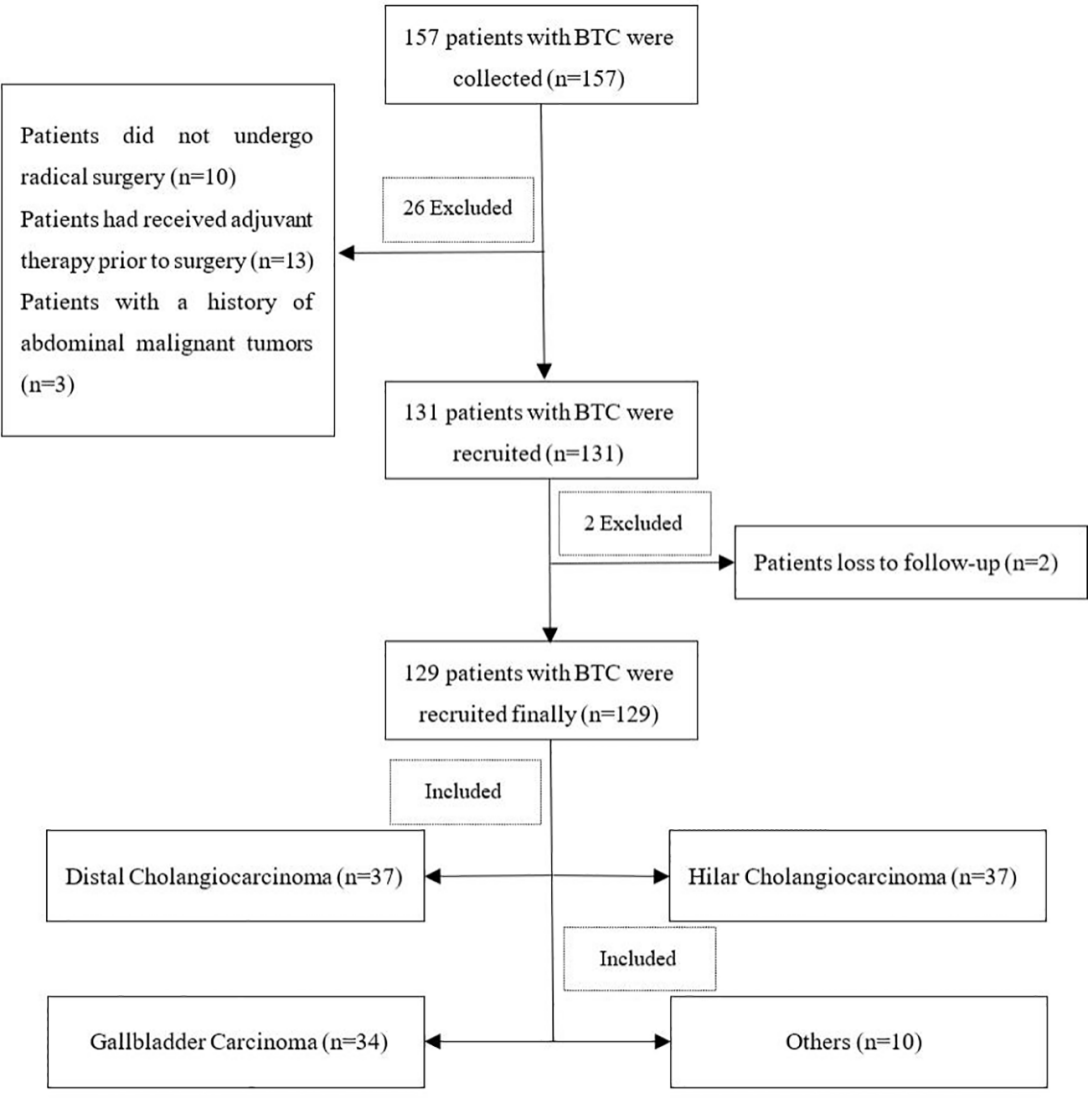
Flowchart of inclusion and exclusion criteria in this study.

Perioperative data from 157 patients with BTC were collected from the database. After excluding three patients with a history of previous abdominal malignant tumors, 10 patients who did not undergo radical surgery, 13 patients who had received neoadjuvant therapy prior to surgery, and two patients lost to follow-up, a total of 129 BTC patients were finally enrolled in this study. Of these 129 patients, there were 37 cases of distal cholangiocarcinoma, 34 cases of gallbladder carcinoma, 48 cases of hilar cholangiocarcinoma, and 10 cases of other malignancies (flowchart is show in **Figure 1**) (four cases of carcinoma of duodenal papilla and six cases of intrahepatic cholangiocarcinoma). Twenty-one patients underwent pancreaticoduodenectomy, including six patients who underwent laparoscopic surgery, six patients underwent robotic operation, and nine patients underwent open surgery. **Table 2** shows the statistical data of patient demographics and preoperative variables comparing CCI< 40 with CCI ≥ 40.

**Table 2:** Regression analysis of demographic data and perioperative factors influence the risk of severe complications.

No.	Variable	Complication comprehensive index		Univariable	Multivariable		
		CCI< 40 (n = 97, 75%)	CCI ≥ 40 (n = 32, 25%)	p	OR	95%CI	p
1	Age, year, n (%)			0.226	0.985	0.931–1.042	0.596
	<70 years	72 (56%)	17 (13%)				
	≥70 years	25 (19%)	15 (12%)				
2	Gender, n (%)			0.575			
	Male	57 (44%)	20 (16%)				
	Female	40 (31%)	12 (9%)				
3	BMI (range)	22.40 (16.89–33.30)	22.93 (18.28–28.08)	0.948			
4	Tumor type, n (%)			0.364			
	Distal cholangiocarcinoma	27 (21%)	10 (8%)				
	Gallbladder carcinoma	28 (22%)	6 (5%)				
	Hilar cholangiocarcinoma	35 (27%)	13 (10%)				
	Others	7 (5%)	3 (2%)				
5	Leucocyte count, 10^9^ (range)	6.00 (3.5–13.10)	6.10 (4–16.60)	0.702			
6	ENBD or PTBD, n (%)	53 (41%)	22 (17%)	0.137	1.247	0.381–4.081	0.715
7	Preoperative TB, μmol/L (range)	33.30 (2.99–239.80)	63.70 (3.10–221.80)	0.253	1.009	0.993–1.025	0.253
8	ΔAlbumin, g/L (range)	6.5 (3–12.1)	7.7 (5.4–11.6)	**0.002**	0.677	0.471–0.975	**0.036**
9	ΔHemoglobin, g/L (range)	3.4 (2.2–5.2)	3.5 (2.4–5.1)	0.076	1.033	0.921–1.158	0.581
10	INR, point (range)	0.99 (0.87–1.42)	1.04 (0.87–1.37)	**<0.001**	0.002	0.000–0.206	**0.009**
11	T stage, n (%)			**<0.001**	15.096	2.117–107.666	**0.007**
	T1–3	95 (73%)	20 (16%)				
	T4	2 (2%)	12 (9%)				
12	Preoperative cholangitis, n (%)	5 (4%)	11 (9%)	**0.01**	0.281	0.031–2.573	0.261
13	Minimally invasive surgery, n (%)	14 (11%)	5 (4%)	0.733			
14	CRS ≥ 1, n (%)	9 (7%)	17 (13%)	**<0.001**	0.137	0.031–0.613	**0.009**

Significant p-values are those<0.05 as indicated in bold.

BMI, body mass index; ENBD, endoscopic nasobiliary drainage; PTBD, percutaneous transhepatic biliary drainage; TB, total bilirubin level; Δ, the reduced values between the first day after surgery and pre-operation; INR, international normalized ratio; CRS, comprehensive risk score.

### Postoperative events

Four patients died after radical surgery at the hospital (two patients due to massive gastrointestinal hemorrhage, one patient due to multiple organ dysfunction syndrome, and one patient due to bile leakage and septic shock). CCI< 40 occurred in 75% (97/129) of patients with moderate or no complications, and severe morbidity was observed in 25% (32/129) of patients with CCI ≥ 40. Radical surgery combined with pancreaticoduodenectomy was performed in 26 patients (20%), and 19 patients (15%) underwent minimally invasive surgery (including seven patients with robotic approach). The complication distribution according to tumor type was 34 patients in gallbladder carcinoma, 37 patients in distal cholangiocarcinoma, 48 patients in hilar cholangiocarcinoma, and 10 patients in others. The Clavien–Dindo classification of complications and their distribution is depicted in **Table 3**.

**Table 3:** Type and grade of postoperative complications according to Clavien–Dindo classification.

Most severe complication	Grade according to Clavien–Dindo								Overall, n (%)
	0	1	2	3a	3b	4a	4b	5	
Without complications	14								14 (11%)
Bile leakage		1	3	3	3				10 (8%)
Acute cholangitis			1	2		2			5 (4%)
Liver failure			2			1	1		4 (3%)
Intra-abdominal fluid collection		3		3	1				7 (5%)
Intra-abdominal abscess		2	3	4	1				10 (8%)
Ascites			2	4					6 (5%)
Intra-abdominal hemorrhage			1	5	4			2	12 (9%)
Intestinal obstruction			1	1	2				4 (3%)
Pleural effusion		1	3	4					8 (6%)
Electrolyte disorder		1	2						3 (2%)
Pulmonary infection		2	8						10 (8%)
Postoperative gastroplegia syndrome		1	2	2					5 (4%)
Septic shock							1	1	2 (1.5%)
Acute renal failure				3		1	1		5 (4%)
Pancreatic fistula			2	2	2				6 (5%)
Liver abscess			1	2					3 (2%)
Duodenal fistula							1		1 (1%)
Atrial fibrillation			3			2			5 (4%)
Anastomotic fistula				2	2				4 (3%)
Multiple organ dysfunction syndrome							2	1	3 (2%)
Chylous fistula			1	1					2 (1.5%)
Total	14	11	35	38	15	6	6	4	129 (100%)

Fourteen perioperative variables (including age, sex, body mass index, tumor type, leukocyte count, ENBD or PTBD performed, preoperative total bilirubin level, Δalbumin (the reduced values between the first day after surgery and preoperation), Δhemoglobin (the reduced values between the first day after surgery and preoperation), international normalized ratio, T stage (UICC of 8th edition), preoperative cholangitis (Tokyo Guidelines 2018), minimally invasive surgery, and CRS ≥1) were assessed for their potential in predicting major complications. Four factors were shown to be significantly associated with severe morbidity in multivariate analysis: CRS ≥ 1 (p = 0.009, 95% CI 0.031–0.613), international normalized ratio (INR) (p = 0.009, 95% CI 0.000–0.206), Δalbumin (p = 0.036, 95% CI 0.471–0.975), and T4 stage (p = 0.007, 95% CI 2.117–107.666). ΔHemoglobin had no statistically significant effect on the probability of severe morbidity, and preoperative cholangitis was thought to be associated with severe morbidity in univariate analyses, but no association was found in multivariate analyses.

**Figure 2:**
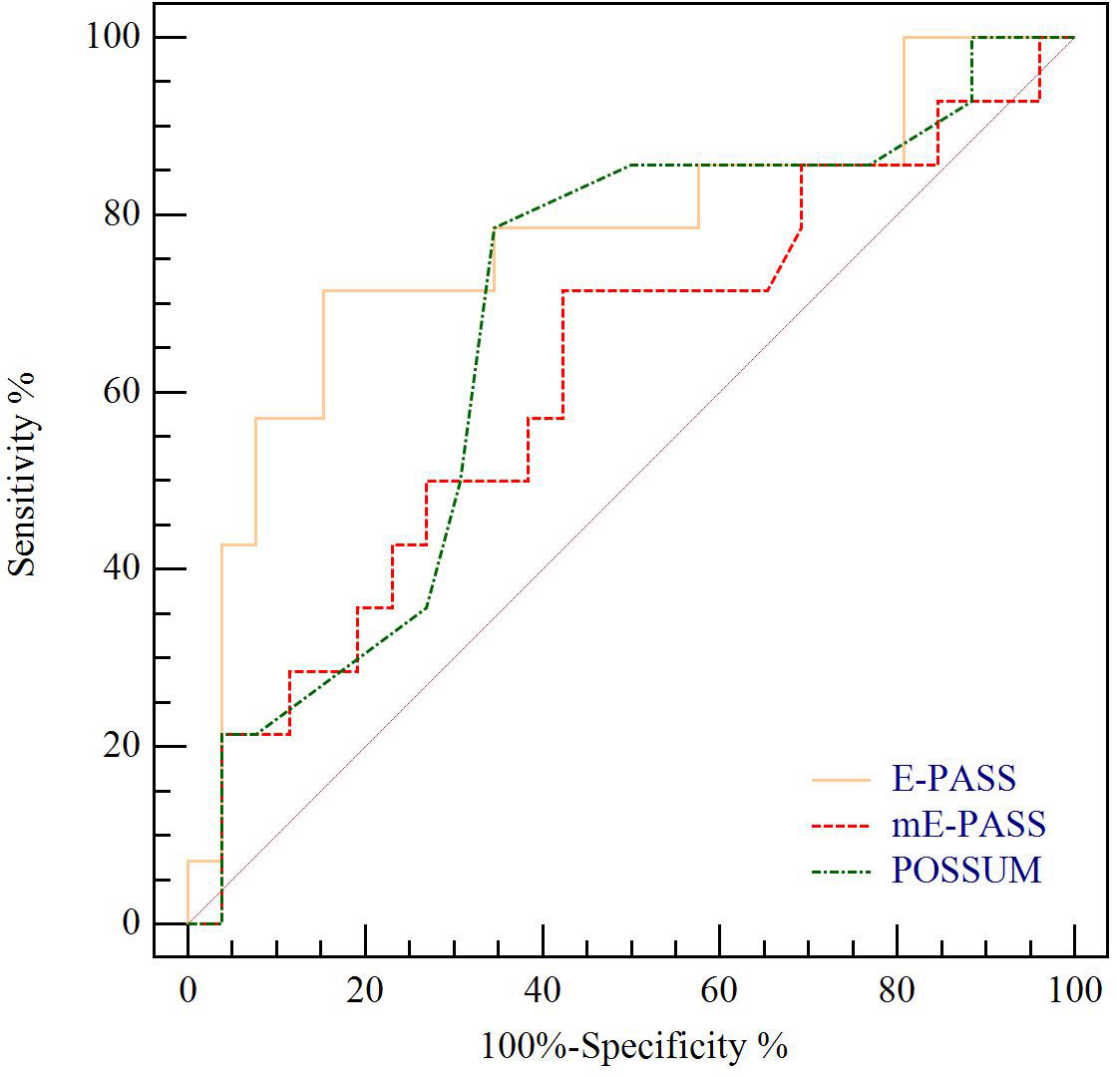
ROC curve analysis of severe morbidity prediction according to CCI in BTC patients with age ≥ 70 years. Yellow line indicates ROC plot for predictive value of E-PASS model alone, AUC 0.772, p = 0.005. Red line indicates ROC plot for predictive value of mE-PASS model alone, AUC 0.620, p = 0.217. Green line indicates ROC plot for predictive value of POSSUM model alone, AUC 0.680, p = 0.063. ROC curve, receiver operating characteristic curve; CCI, Comprehensive Complication Index; AUC, the area under the curve; E-PASS, Estimation of Physiologic Ability and Surgical Stress System; mE-PASS, modified Estimation of Physiologic Ability and Surgical Stress System; POSSUM, Physiological and Operative Severity Score for the Enumeration of Mortality and Morbidity.

**Figure 3:**
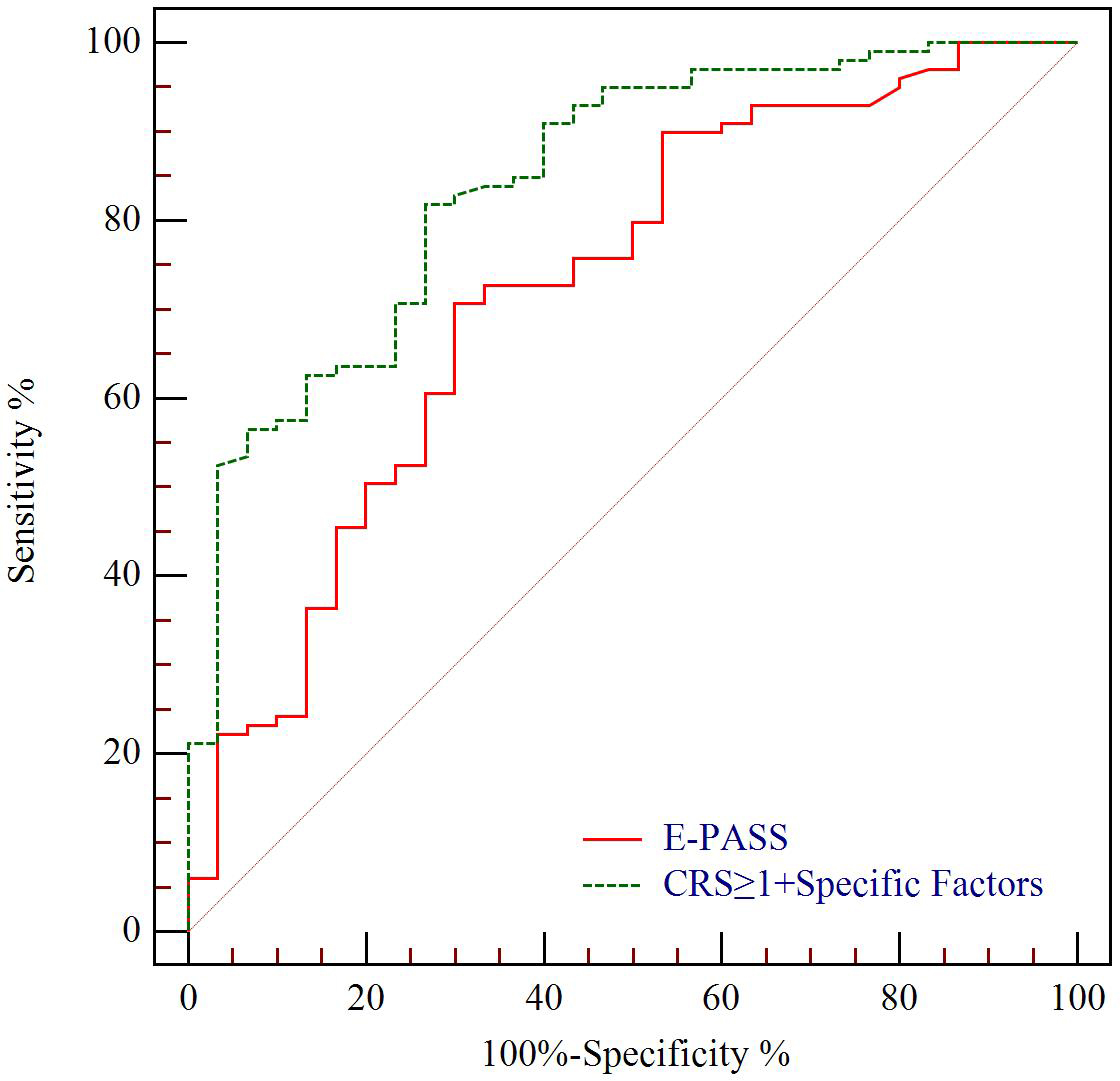
ROC curve analysis of severe morbidity prediction according to CCI in all patients. Green line indicates ROC plot for predictive value of CRS ≥ 1 combined with disease-specific factors including T stage, Δalbumin, and INR, AUC 0.844. Red line indicates ROC plot for predictive value of E-PASS model alone, AUC 0.724 (p = 0.0375). CCI, complication comprehensive index; CRS, comprehensive risk score; Δalbumin, the reduced values between the first day after surgery and pre-operation; INR, international normalized ratio.

**Figure 4:**
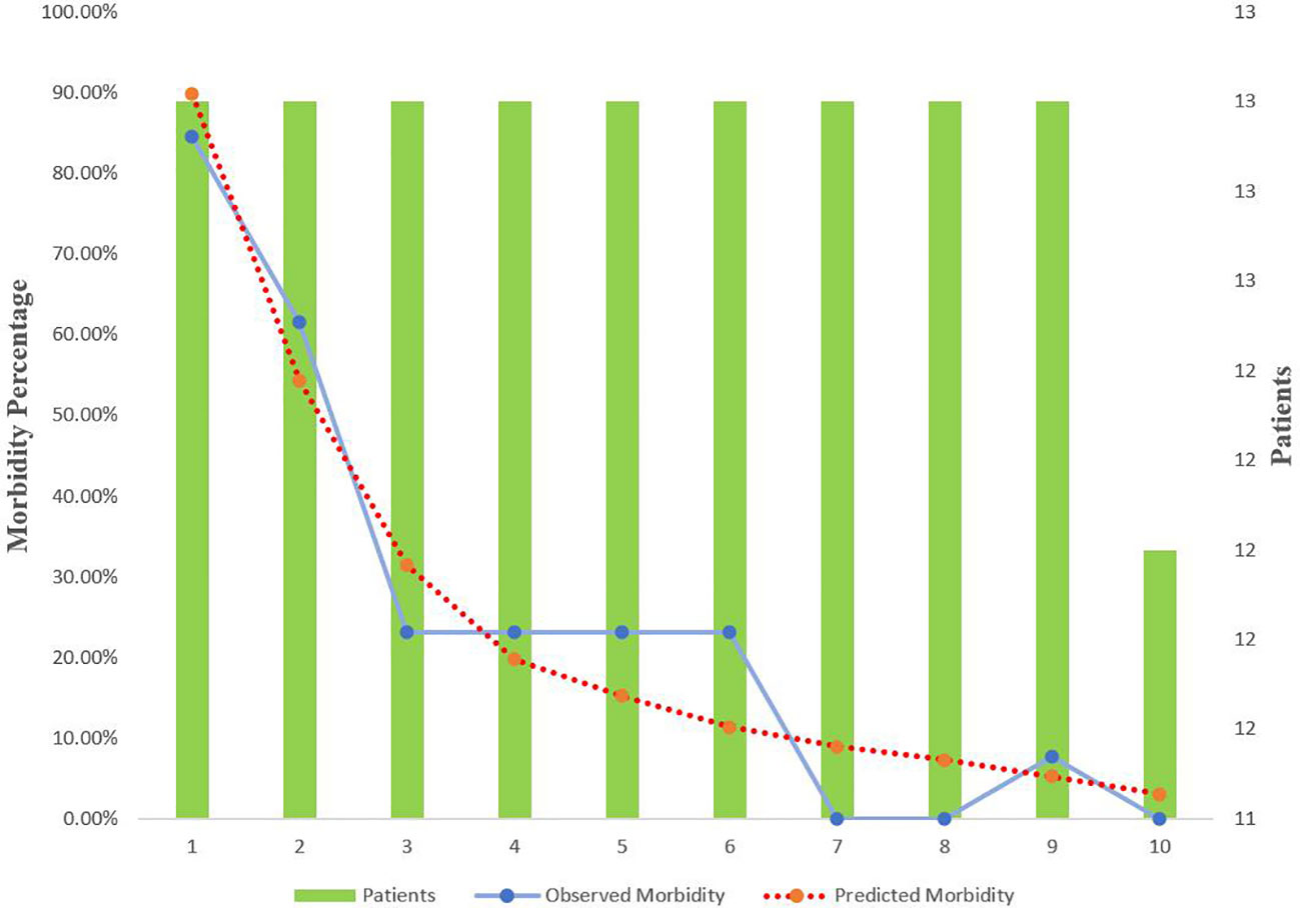
Calibration curve for CRS ≥ 1 + specific factors score for BTC patients with radical operation. Specific factors include T stage, Δalbumin (the reduced values between the first day after surgery and pre-operation), and international normalized ratio. CRS, comprehensive risk score; BTC, biliary tract cancer.

The predictive value for E-PASS, mE-PASS, and POSSUM in all patients was evaluated first using ROC plots. Interestingly, the present study discovered that the mE-PASS and POSSUM models do not accurately predict the occurrence of severe morbidity among patients over the age of 70, and the AUC of each model applied in selected patients was 0.772 for E-PASS (p = 0.005), 0.620 for mE-PASS (p = 0.217), and 0.680 for POSSUM (p = 0.068) (**Figure 2**). Second, in all patients, the predictive efficacy of CRS ≥ 1 with perioperative variables was compared to that of the E-PASS alone, and there was a significant difference, with AUC values of 0.844 and 0.724, respectively (p = 0.0375). This finding revealed that CRS ≥ 1 combined with perioperative variables had a better predictive effect than the E-PASS model alone (**Figure 3**). CRS ≥ 1 in combination with disease-specific factors demonstrated good calibration by Hosmer and Lemeshow analysis, p ≥ 0.05 (**Figure 4**).

## Discussion

This is the first study to evaluate how perioperative risk variables combined with risk models might predict postoperative morbidity in BTC patients. Advances in radical surgical techniques and improvements in perioperative treatment have assisted in minimizing the risks associated with surgery for patients in recent years. However, due to the complexity of the operation, the incidence of postoperative complications is still high, up to 35%–68% (22, 23). Postoperative morbidity may be related to three factors: the pressure on the surgical team, the patient’s physical condition, and surgical stress. Because the skillfulness of the surgical team at this institution has been steady for some time, all patient information in this study was collected from the same surgical team to lower the influences of the pressure of the surgical team. The influences of other factors, including the patient’s physical condition and surgical stress, could be associated with postoperative complications. This study found that surgeons should extensively evaluate the risk of operation based on each patient’s physical state to choose the appropriate opportunity for an operation.

The CDC insufficiently handles overall postoperative events in patients since patients may suffer a variety of moderate and severe postoperative morbidities. As a result, the CCI may provide more precise information on the prevalence of postoperative adverse events because the CCI calculates all morbidity in one patient. Previous research has confirmed the predictive value of CCI in quantifying the probability of major complications in BTC patients after radical resection (9), and previous studies have also shown that E-PASS (24), mE-PASS (7), and POSSUM (25) could predict the occurrence of severe morbidity in general surgery. As a result, these grading methods and risk models were used in this research. Unlike the SSS of the E-PASS model, which can precisely calculate surgical scores of operation time, blood loss, and the length of the skin incision, the SSSf of mE-PASS and OS of POSSUM can only estimate surgical scores roughly. The present study found that the mE-PASS and POSSUM models do not effectively forecast the occurrence of severe morbidity in older patients (age ≥ 70). One possible explanation for the difference in findings was that blood loss and skin incisions have a significant impact on older people. Thus, it is a better choice to utilize the E-PASS model to predict severe morbidity in patients over the age of 70. Furthermore, the mE-PASS and POSSUM were created for surgical auditing purposes solely, not for surgical decision-making. The utilization of E-PASS, however, has the possibility of playing a role not only in surgical auditing but also in clinical choice both between and within individual practices (26).

Cholangitis was confirmed as a quite important factor for major morbidity according to the Tokyo Guidelines 2018 (21). In the univariable analysis, preoperative cholangitis was related to severe complications, but not in the multivariable analysis. The possible reason was assumed to be that biliary drainage was applied to address preoperative cholangitis. Previous studies have also shown that patients with cholangitis are more likely to suffer severe complications after surgery than those who undergo surgery after their cholangitis is cured (27, 28). Therefore, biliary drainage should be performed as soon as possible in patients with preoperative cholangitis or biliary obstruction, and the operation should be postponed until preoperative cholangitis is resolved.

Albumin, as an acute-phase protein that responds to systemic inflammation and surgical trauma, can maintain blood osmotic pressure and transport amino acids, medicines, hormones, and other macromolecular compounds in the blood (29, 30). According to previous studies, hypoalbuminemia is closely related to the occurrence of a variety of postoperative morbidities (such as systemic infection), suggesting that severe albumin loss may be a predictor of the occurrence of postoperative complications in patients with BTC (31). As we all know, albumin plays an important role in the homeostasis of the body, and hypoproteinemia, as a sign of malnutrition, is a consequence of suppressed or increased loss (32). In this work, we found that the presence of albumin depletion is an independent risk factor for predicting the occurrence of severe morbidity. Patients with biliary tract cancers have more surgical trauma and albumin loss following radical surgery; if hypoproteinemia continues over an extended period of time after the operation, it will lead to an increased adverse prognosis, such as bile leakage, sepsis, or even death (30). Therefore, patients with severe albumin loss in the early postoperative period should obtain suitable albumin infusions and closely monitor their vital signs, which can be paired with additional laboratory tests or imaging results if appropriate.

The present study indicated a good predictive value for the combined application of perioperative variables and CRS ≥ 1, which was not found for applying CRS alone. However, the excellent predictive value of grading approaches has not yet been found, and a new prognostic score was not proposed since it would be biased due to the small sample size.

There are some drawbacks to this study. First, the number of enrolled patients was small. Increasing the number of cases may improve the accuracy and reliability of the outcomes. Second, this study only collected follow-up data within 3 months of the operation, which may have omitted cases of adverse outcomes. Finally, the main content of this study is the predictive value of the risk model combining specific risk factors in all patients with BTC; however, different types of biliary tract tumors have different heterogeneity, and in-depth studies on different types of biliary tract tumors are needed to further verify the application value of this prediction method in different types of biliary tract tumors.

## Conclusion

According to the findings of this study, mE-PASS and POSSUM are ineffective for predicting postoperative morbidity in patients over the age of 70. Meanwhile, the prediction accuracy of specific factors combined with E-PASS is superior to E-PASS alone. The most essential perioperative variables for major complications were found as T stage, albumin decrease value, and INR. However, future advancements are required because none of these grading approaches produced an AUC value larger than 0.90 for procedures differing in severity.

## Data availability statement

The raw data supporting the conclusions of this article will be made available by the authors, without undue reservation.

## Ethics statement

The requirement of ethical approval was waived by The ethics committee of Jinling Hospital, Medical School of Nanjing University for the studies on humans because The ethical approval of this work were unnecessary from the ethics committee of Jinling Hospital, Medical School of Nanjing University. The studies were conducted in accordance with the local legislation and institutional requirements. Written informed consent for participation was not required from the participants or the participants’ legal guardians/next of kin in accordance with the national legislation and institutional requirements. The human samples used in this study were acquired from primarily isolated as part of your previous study for which ethical approval was obtained. Informed consent was obtained from all individual participants included in the study.

## Author contributions

BY: Writing – original draft. JX: Writing – original draft. KX: Data curation, Formal analysis, Writing – original draft. ZH: Formal analysis, Writing – original draft. YL: Data curation, Writing – original draft. ZC: Writing – review & editing. WJ: Writing – review & editing.
